# Chicago Medical Society

**Published:** 1868-07

**Authors:** 


### CHICAGO MEDICAL SOCIETY.

Friday, May 22, 1868.

The regular meeting of the Chicago Medical Society was called to order by Dr. Wickersham, who was chosen President, *pro tem.* The Secretary read the minutes of the last meeting, which were approved.

Under the call for pathological specimens, Dr. Holmes presented two cataracts, one being of unusual large size; also a modified and improved cataract knife.

Dr. Durham reported the case of a lady whom he had been attending for some time, who had chronic diarrhœa. The patient never had much cough. A *post mortem* revealed extensive miliary tubercle in both lungs, and, also, ulceration of the intestine; specimens of each being exhibited to the Society.

Dr. Davis remarked that there had probably been a pleuro-pneumonic inflammation previous to death. Did not think there was a deposit of tubercle in the menentery. Said he had seen a goodly number of cases of chronic diarrhœa with pulmonary tuberculosis, in which there was no noticeable cough if the diarrhœa were not controlled; but if the diarrhœa was checked for two or three weeks, a cough would supervene, with muco-purulent expectoration. A *post mortem* revealing, in either case, pulmonary tuberculosis. Recommended that Dr. Durham examine the specimens under the microscope, and report the same to the Society.

Under reports of cases, Dr. Wanzer reported the case of a man who fell and split his nose and lip, the wound filling with splinters and sand, which, however, healed by granulations; but his nose was decidedly one-sided, which, nevertheless, he righted by an operation. He also reported the case of a little boy, 5 years old, who had his leg crushed by a wagon wheel. Stated that the bone was denuded for some three inches below the patella, and the inflammation was very great, and asked the Society for advice in the matter.

Dr. Davis did not think any advice could be given, without seeing the patient.

Dr. Wickersham said that he had been informed that the cattle in Sangamon Co. were dying off at the rate of 75 per day from a disease, as yet unknown, and he thought it the duty of the Society, to discover, if possible, what this disease was, and the treatment.

Dr. Davis regarded the report as well worthy the attention of the Society, and recommended that a committee be appointed to investigate the matter, should the disease continue to extend.

On motion of the President, the following committee was appointed: Drs. Wickersham, Davis, Paoli, Bogue, and Durham, whose duty it should be to report from time to time, if deemed necessary.

Dr. Davis recommended that the Society only meet once a month during the months June, July, and August, and that on the first Friday in each month. Motion carried.

Society adjourned.

---

Friday, May 29, 1868.

The regular meeting of the Chicago Medical Society was called to order by the President, Dr. Marguerat. The Secretary read the proceedings of the last meeting, which were duly approved and ordered to be placed on file.

Dr. A. H. Gray, who was recommended for membership at the last meeting, and favorably reported on by the Board of Censors, was balloted for, and duly elected a member of the Society.

The Society then proceeded to the discussion of the regular subject, chosen at a previous meeting, *viz.:* "Treatment in cases of disproportion of the size of the fœtal head to the pelvis."

The discussion was commenced by Dr. John Reed, and participated in by Drs. Paoli, Davis, Bevan, Wickersham, Holmes, and Loverin. No new facts or modes of practice were developed, and hence we omit the usual synopsis.

The subject chosen for discussion on the first Friday in July was, "What effect would ice cut from the basin on the lake shore have upon the health of the people using it for drinking purposes?"

Dr. Bevan asked between what hours constituted a night visit? It was decided from 10 P.M. to 6 A.M., when the the physician was allowed double fees.

The Society then adjourned.

---

Friday, June 5, 1868.

The regular meeting of the Chicago Medical Society was called to order by the President, Dr. Marguerat, and the proceedings of the previous meeting were read by the Secretary and approved.

Under the call for committee reports, Dr. Durham, in behalf of the committee appointed to investigate the cattle disease, said that he had been furnished with some information on the subject, and exhibited to the Society a letter from Dr. Geo. B. Allen, of Springfield, in which he regarded this disease as a pleuro-pneumonia. He also read an article which was published in the *Springfield Journal*, by the same author. Stated that he has been informed by Dr. Janes that the disease has entirely abated.

Under call for pathological specimens, Dr. Bogue presented a portion of the vertebræ of a man who had died in the County Hospital, from fracture of the twelfth dorsal vertebra, caused by being run over and doubled up by a hand-car. He was admitted to the Hospital at the time of the accident, April 14, with complete loss of sensation and motion of the lower extremities, but no pain. May 4, bed-sores and marked deformity at the point of injury. May 9, bed-sores sloughing; a thin, fetid discharge from bowels constantly. On May 31, the patient died from exhaustion. Stated that below the seat of injury the temperature was about two degrees higher than above. *Post mortem* revealed a complete fracture of vertebra with the last ribs twisted from their attachment, and the cord softened about the fracture. In this case, there was no counter-extension employed, and only the palliative remedies administered.

The practicability of opening to the cord, by means of a trephine, was discussed by Drs. Lyman, Holmes, Paoli, Bogue, Durham, and Wickersham; the latter gentleman believing that cases of fracture of the vertebræ should not be experimented on by this uncertain means, thereby avoiding suits for malpractice.

Dr. Bogue believes that where there is no mobility nor crepitus, it is useless to operate by trephining.

Dr. Lyman thinks if 10 per cent of these cases can be saved, it will justify the operation.

Under the head of cases, Dr. Wickersham reported the case of a man who had a soft chancre, nine or ten months ago, who was successfully treated. A short time since, he had an eruption, resembling secondary disease, come out on the forehead. This eruption occurred some 36 or 48 hours after the patient had commenced wearing red flannel shirts, and upon discontinuing wearing them the eruption disappeared in 48 hours. He believes physicians, generally, to be too hasty in their diagnosis, frequently telling patients they have constitutional disease, when there is no foundation for the statement. He also related a case, where a lady was troubled with a similar eruption from eating strawberries, the eruption always occurring in strawberry season.

Dr. Loverin says he has cases of this rash, from the wearing of red flannel, extending over the body.

Dr. Durham related the case of a man, who fell on the ice while crossing the river at Atchison, Kansas, early last winter. He did not fall entirely down, but merely slipped, saving himself by allowing his weight to come on one hand. The first symptoms were faintness and a feeling of nausea, he not being able to move for upwards of half an hour, during which he remained lying on the ice. Finally, he arose, feeling pretty well, and resumed his journey to Valparaiso, Ind., where he resided. States that in four months his weight was reduced from 198 to 140 pounds, but he now weighs about 160 pounds. On examination, he found pulse 83, shortness of breath, and retention of urine. Over the third, fourth, and fifth dorsal vertebræ great tenderness on pressure, but, to all appearances, no displacement or abnormal appearance of the spine, there being only this excessive hyperæsthesia on the right side. Amount of urine very great.

Dr. Lyman thought this case of much interest, and hoped Dr. Durham would have it published, as it is undoubtedly an injury of the spine from a comparatively slight cause.

Dr. Paoli asked as to the possibility of this case being one of malingering; citing one in his own practice.

The discussion was also participated in by Drs. Bogue and Loverin.

Dr. Holmes related a case, where the use of chloroform was followed by dangerous symptoms, the pulse falling from 120 to 46 beats per minute, and then ceasing entirely. Circulation was established by placing the patient's head down, at an angle of 45°. He also stated that a similar case, last winter, speedily recovered under the same treatment.

The Society then adjourned until the first Friday in July.

